# Direct sample introduction GC-MS/MS for quantification of organic chemicals in mammalian tissues and blood extracted with polymers without clean-up

**DOI:** 10.1007/s00216-020-02864-6

**Published:** 2020-08-15

**Authors:** Andreas Baumer, Beate I. Escher, Julia Landmann, Nadin Ulrich

**Affiliations:** 1 Department Cell Toxicology, Helmholtz Centre for Environmental Research–UFZ, 04318 Leipzig, Germany; 2 Environmental Toxicology, Centre for Applied Geoscience, Eberhard Karls University Tübingen, 72074 Tübingen, Germany; 3 Institute of Anatomy, University of Leipzig, 04103 Leipzig, Germany; 4 Department Analytical Environmental Chemistry, Helmholtz Centre for Environmental Research–UFZ, 04318 Leipzig, Germany

**Keywords:** Mammalian tissues, Direct sample introduction GC-MS/MS, Passive sampling, PDMS, Co-extracted matrix, Reduction of matrix effects

## Abstract

Solvent extracts of mammalian tissues and blood contain a large amount of co-extracted matrix components, in particular lipids, which can adversely affect instrumental analysis. Clean-up typically degrades non-persistent chemicals. Alternatively, passive sampling with the polymer polydimethylsiloxane (PDMS) has been used for a comprehensive extraction from tissue without altering the mixture composition. Despite a smaller fraction of matrix being co-extracted by PDMS than by solvent extraction, direct analysis of PDMS extracts was only possible with direct sample introduction (DSI) GC-MS/MS, which prevented co-extracted matrix components entering the system. Limits of quantitation (LOQ) ranged from 4 to 20 pg μL^−1^ ethyl acetate (PDMS extract) for pesticides and persistent organic pollutants (POPs). The group of organophosphorus flame retardants showed higher LOQs up to 107 pg μL^−1^ due to sorption to active sites at the injection system. Intraday precision ranged between 1 and 10%, while the range of interday precision was between 1 and 18% depending on the analyte. The method was developed using pork liver, brain, and fat as well as blood and was then applied to analyze human post-mortem tissues where polychlorinated biphenyls (PCBs) as well as dichlorodiphenyltrichloroethane (DDT) and DDT metabolites were detected.

**Electronic supplementary material:**

The online version of this article (10.1007/s00216-020-02864-6) contains supplementary material, which is available to authorized users.

**Figure:**
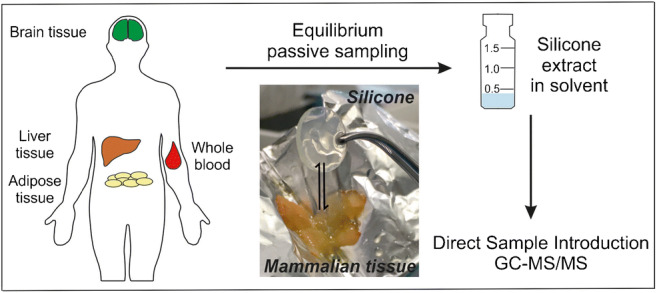
Graphical abstract

## Introduction

Organisms are exposed to a variety of chemicals that are present in environmental matrices like air, water, and soil. Especially hydrophobic organic chemicals (HOCs) tend to bioaccumulate via food webs in biota tissues due to their high lipid solubility and persistence against degradation [1]. One example of HOCs are persistent organic pollutants (POPs) like polychlorinated biphenyls (PCBs) or organochlorine pesticides (OCPs) which have been measured in biota samples from the aquatic environment [2], bird eggs [3], polar bears [4], and in human tissues like liver, kidney, brain, and adipose tissue [5–7]. Since human tissues are generally not easily accessible for the analysis of POP concentrations, blood is often used as sample matrix for the investigation of human exposure [8]. As exposure of humans to POPs has decreased due to restrictions in the last decades, biomonitoring studies are now focusing on other bioaccumulative compounds like flame retardants, plasticizers, environmental phenols, or fungicides [9]. These compounds are not as persistent as POPs and hence have lower bioaccumulation factors, but the elimination from specimens can still require weeks up to months [9].

Most analyses of biological tissues have been carried out using solvent extraction methods followed by various clean-up procedures in order to reduce the amount of co-extracted matrix components prior to GC-MS measurements [10–12]. These extractions have a high solvent consumption as well as often time-consuming and labor-intensive purification steps of sample extracts. Most of the applied clean-up procedures degrade non-persistent contaminants. Equilibrium passive sampling with polydimethylsiloxane (PDMS) can be applied to complex biota matrices [13] without altering the mixture composition, including all HOCs [14, 15]. This extraction method was applied to the analysis of environmental contaminants in various complex biota matrices, e.g., fish [13, 16, 17], eel [18], blubber [19, 20], blood plasma [21], urine [22], and whole blood [22, 23]. HOCs are selectively taken up by the PDMS, because only uncharged molecules are able to diffuse into the polymer, whereas the uptake of proteins, salts, or metals is hindered [23]. Thus, equilibrium passive sampling with PDMS results in less co-extracted material compared to raw extracts of traditional solvent extraction methods. In case of dugong blubber with a lipid content of 85% (w/w), only approximately 4 mg lipids per g PDMS were co-extracted from 500 mg tissue (corresponding to 0.8% of co-extracted lipids) at equilibrium [19, 24]. Another study investigated the partitioning of POPs between PDMS and pure oils and reported an average co-extracted amount of lipids of 0.9% [25].

Even this small amount of co-extracted lipids can contaminate sensitive analytical instruments, such as GC-MS systems. Using solvent injection techniques employing split/splitless (SSL) injectors, non-volatile co-extracted matrix components (e.g., triglycerides) can build up in the injection port and at the front of the capillary column generating active sites which leads to peak broadening or peak tailing, loss of sensitivity, and matrix-induced peak enhancement, also known as matrix effects [26, 27]. Analytical results are severely affected and time-intensive maintenance of the GC system and ion source has to be conducted. Although sample extracts contain only small amount of co-extracted matrix, there is still a need of extract purification, typically by the addition of sulfuric acid in order to digest lipids followed by further treatment using a Florisil® column with drying agents for elimination of water [13, 19, 23, 28]. If POPs like PCBs are analyzed, which are not degraded by the treatment with sulfuric acid, no severe loss of these analytes occurs [28]. If the analysis includes pH labile analytes, other non-aggressive clean-up procedures like gel permeation chromatography (GPC) [29–31], solid-phase extraction (SPE) [31–33], dispersive solid-phase extraction (dSPE) [34–36], or low temperature precipitation (freeze out of lipids) [36–38] are employed either as single clean-up step or in combination.

Analysis of raw sample extracts using GC circumventing the clean-up steps mentioned above can be conducted by the principle of direct sample introduction (DSI), which was reported by Amirav and Dagan [39], and is also known as difficult matrix injection (DMI). Briefly, raw extracts obtained from solvent extractions, liquid samples like urine and oil, or even solid samples are placed in disposable micro vials (μ-vials), which are introduced to the programmable temperature vaporizing (PTV) injector at low temperatures. After heating to a temperature required for evaporation of the (semi-) volatile analytes, the non-volatile matrix components remain inside the μ-vial, whereas the analytes are refocused on front of the cold analytical column. The μ-vial with the remaining matrix is exchanged during chromatographic separation resulting in less GC system maintenance and analytical interferences. The DSI approach was evaluated in different matrices like fruits [40–42], vegetables [42, 43], cereals [42, 44], eggs [45], and oils [46, 47].

The aim of our work was to develop an analytical method for liver, brain, and adipose tissue as well as for whole blood and to investigate the chemical burden of human tissues and blood. We selected 27 chemicals including PCB congeners, DDT and its metabolites dichlorodiphenyldichloroethane (DDD) and dichlorodiphenyldichloroethylene (DDE), pesticides like atrazine (ATZ), metolachlor (MTC), and chlorpyrifos-methyl (CPM) as well as organophosphorus flame retardants like triphenyl phosphate (TPP) and tris(2-chloroethyl) phosphate (TCEP). To achieve this aim, we combined the equilibrium passive sampling with PDMS and the DSI approach employing thermodesorption to provide a method for unbiased analysis of PDMS extracts with an application to pork and human tissues covering a broad range of persistent and non-persistent hydrophobic chemicals with different functional groups and physicochemical properties.

Typically, the analysis of the analytes extracted with PDMS is carried out by direct thermodesorption of the silicone sampler. Although the direct analysis saves time, the analysis of the corresponding solvent extract has advantages because it allows multiple detection methods, such as different ionization modes in GC or analysis with liquid chromatography. Furthermore, repeated analysis of the same sample is possible (e.g., in case of instrumental error). Importantly, for future studies, the chemical analysis can be combined with toxicological screening using in vitro bioassays [48]. To allow parallel application of analytical and bioanalytical methods, we applied higher volumes of PDMS, which also allows higher enrichment of the samples because the partition coefficients between tissue and PDMS are low [25] and will not enrich chemicals of low concentration sufficiently for detection.

## Experimental section

### Chemicals

Chemicals included in this study with their CAS number, abbreviation, purity, and supplier can be found in the Electronic Supplementary Material (ESM, Section S1, Table S1). For the determination of total lipid content of biota samples, 1-palmitoyl-2-oleoyl-sn-glycero-3-phosphocholine (POPC, Avanti Polar, Alabaster, AL, USA) and triolein (Sigma-Aldrich, Taufkirchen, Germany) were used as standards. Bovine serum albumin (BSA) (Sigma-Aldrich, Taufkirchen, Germany) was used as negative control. Ethyl acetate (EA), dichloromethane (DCM), and methanol (MeOH) were purchased in GC grade and cyclohexane (CH) and isopropanol (IPA) were purchased in LC grade, all from Merck (Darmstadt, Germany). Polydimethylsiloxane (PDMS, SSP-M823, Special Silicone Products, Ballston, USA) sheets (approximately 30 cm × 30 cm) with thicknesses of 1 mm, 0.6 mm, and 0.3 mm, and a density of 1.17 g cm^−3^ were provided by Shielding Solutions (Great Notley, Great Britain).

### Tissue and blood samples

Pork liver, brain, and fat were bought at a local butchery. Whole blood from pig (containing 1.5 mg ethylenediaminetetraacetic acid dipotassium salt (K_2_EDTA) per mL whole blood) was obtained from Fiebig-Nährstofftechnik (Fiebig-Nährstofftechnik GbR, Idstein–Niederauroff, Germany).

Human post-mortem liver, brain, blood, and (abdominal) adipose tissue were obtained from one body donor. Being part of the body donor program regulated by the Saxonian Death and Funeral Act of 1994 (third section, paragraph 18 item 8), institutional approval for the use of the post-mortem tissues of human body donors was obtained from the Institute of Anatomy, University of Leipzig. All authors declare that all experiments were conducted according to the principles of the Declaration of Helsinki [49]. Blood from a volunteer (29 years, male) was collected under medical supervision, stored in a commercially available blood donation tube containing K_2_EDTA as anticoagulant yielding a final concentration of 1.8 mg K_2_EDTA per mL whole blood (BD Vacutainer, BD Diagnostics, Heidelberg, Germany). Blood samples obtained from the body donor showed coagulation and progressive hemolysis and could not be used for the analysis. All tissues were homogenized using a blender (B-400, BÜCHI Labortechnik AG, Switzerland). Homogenized tissues as well as whole blood samples were stored at − 20 °C until analysis.

### Determination of total lipid content

Determination of total lipids was carried out gravimetrically after solvent extraction according to Smedes [50] with modifications for the use of small amounts of tissues (50 to 500 mg) outlined in the ESM (Section S2).

### Preparation of PDMS

PDMS was cleaned by Soxhlet extraction with EA for the duration of 24 h in order to remove residual impurities and monomers. Cleaned PDMS was stored in brown DURAN® bottles covered with fresh EA until usage at room temperature. Before immersion into tissue or blood, PDMS was air-dried for at least 2 h to ensure complete evaporation of remaining EA.

### Passive sampling of tissues and blood

A microbalance was used to determine the initial mass of air-dried PDMS in order to monitor the mass gain after passive sampling was completed. Pre-weighted PDMS (approximately 125–400 mg) was carefully immersed into the homogenized tissues or blood. The PDMS weights were tracked individually through all experiments. Equilibrium in passive sampling with PDMS was attained even for analytes with slow uptake kinetics after 168 h for all matrices and was assessed either with time series experiments or with silicone of multiple thicknesses [13] (data not shown). All passive sampling experiments were carried out at 4–8 °C to slow down tissue decay during sampling time.

Three different experimental set-ups depending on the lipid content of the sample were carried out in order to reach equilibrium. (1) Static sampling experiments for fat tissue was carried out with PDMS of 1 mm thickness, which was cut in circular discs with a diameter of 12 mm yielding a weight of approximately 125 mg PDMS per disc. The PDMS disc was sandwiched between two tissue layers, which consisted of 200 mg adipose tissue each. (2) Dynamic sampling experiments for liver and brain tissue were performed with 3 g homogenized tissue in 4 mL vials employing silicone strips of approximately 330 mg PDMS (55 × 5 × 1 mm), which served as stirrer and sampler simultaneously avoiding sample depletion occurring during passive sampling of lean tissues [13]. (3) For blood, 2 mL were extracted with approximately 400 mg PDMS (60 × 10 × 0.6 mm). The PDMS strips were fixed in a 4 mL vial that was sealed with a cap containing a PTFE septum after the blood sample was added. For extraction, the vial was placed on a roller mixer with a speed of 10 rpm because foam formation was observed at higher rotation speed. With the selected rotation speed, blood was well mixed as the wings formed by the tightly fixed PDMS dipped in the blood with every rotation of the vial.

All PDMS to tissue ratios used in the passive sampling experiments were calculated based on the negligible depletion criterion [51]. Since blood was expected to have a lower concentration of target chemicals compared to solid tissues such as liver [35], a high PDMS to blood ratio was used to ensure a nearly exhaustive extraction (mass transfer of 60–80%) according to Jin et al. [23].

### PDMS extraction

After equilibrium was attained after 7 days, the PDMS was removed from the tissue. Adhering tissue or blood on the PDMS surface was wiped off with lint-free paper wipes. The PDMS was dipped into water, dried with lint-free paper wipes, and weighed. The mass gain caused by co-extracted matrix components of the different tissues was recorded. Extraction of PDMS was performed twice with approx. 1 mL EA per 0.1 g PDMS for 2 h on a roller mixer. The combined extracts were transferred to a 1.5 mL vial, blown down under a gentle stream of nitrogen, and dissolved in 50 μL EA or spiked with 50 μL of an appropriate analyte solution either for calibration or validation (see below). The samples were stored in the freezer at − 20 °C until analysis.

### Preparation of standard and spiking solutions

Calibration mixtures of all analytes (ESM, Table S1) were prepared in concentrations of 1, 5, 10, 30, 80, 250 and 750 pg μL^−1^ in EA. Matrix extracts after PDMS sampling with isotopically labeled internal standard solution were used as blanks. The internal standard solution for calibration and also quantitation of human samples consisted of ^13^*C*_12_-PCB congeners 28, 52, 101, 118, 138, 153 and 180, ^13^*C*_3_-atrazine, chlorpyrifos-methyl-*d*_6_, chlorpyrifos-ethyl-*d*_10_, diazinon-*d*_10_, tris-chloroethyl phosphate-*d*_12_, triphenyl phosphate-*d*_15_, DDT-*d*_8_, tributyl phosphate-*d*_27_ and metolachlor-*d*_6_. The concentration in the final mixture (50 μL EA) was 50 pg μL^−1^ for each of the labeled PCB congeners and between 100 and 400 pg μL^−1^ for the other labeled compounds depending on the response with sample matrix. Two different spike mixtures for assessing the intraday and interday precision of the method were prepared to yield low (20 pg μL^−1^, Q_low_) and high (500 pg μL^−1^, Q_high_) concentrations of all analytes in the final matrix extracts.

### Instrumental analysis and direct sample introduction

GC-MS/MS analysis was carried out using an Agilent 7890 GC system with 7010 Triple Quadrupole MS (Agilent Technologies, USA) with a High Efficiency Source (HES) applying an EI energy of 70 eV. A Thermal Desorption Unit (TDU 2) combined with a Cold Injection System (CIS 4) PTV injector (GERSTEL GmbH, Mülheim a. d. Ruhr, Germany) was used for injection. A volume of 1 μL sample extract was injected into the TDU tubes including μ-vials. Maintenance of consumables involved in the DSI process is described in the ESM, Section S3. TDU was operated in splitless mode allowing an efficient transfer to the CIS. The initial temperature of the TDU program was 30 °C and raised to 300 °C at 720 °C min^−1^ (3 min). The helium flow was kept at 50 mL min^−1^ until thermodesorption cycle was completed. The transfer line of the TDU was set to 300 °C. The CIS was operated in solvent vent mode with an empty baffled liner. During thermal desorption, the CIS temperature was set to − 30 °C for cryofocusing. After finishing the thermal desorption cycle, the temperature of the injector was raised to 300 °C at 12 °C s^−1^ (3 min) ensuring a complete transfer of the analytes to the column.

Chromatographic separation of the analytes was achieved on a HP5-MS UI® capillary column (30 m length, 0.25 μm i.d., 0.25 μm film thickness, J&W Scientific, USA). The oven was programmed as follows: 60 °C (3 min) to 210 °C at 30 °C min^−1^ (5 min), to 240 °C at 3 °C min^−1^ and finally to 300 °C at 40 °C min^−1^ (5 min). The run time of the GC oven program was 26 min. Total run time for processing one sample including the exchange of thermal desorption liner, thermal desorption process, and chromatographic separation was 37 min. Helium (6.0 purity) was used as carrier gas in constant flow mode at 1.3 mL min^−1^ and the solvent delay was set at 6.3 min. The MS transfer line was kept at 250 °C, the ion source at 230 °C, and both quadrupoles were operated at 150 °C. Nitrogen was used as collision gas at a flow of 1.5 mL min^−1^. Helium was used as quench gas at 2.25 ml min^−1^. Selective and sensitive mass transitions under specific collision energies (CEs) were determined for each analyte (ESM, Table S2).

After every injection, the syringe was rinsed eight times each with CH followed by EA preventing clogging and sample carryover which may have been caused by remaining matrix components in the syringe plunger. MassHunter Workstation Software–Data Acquisition (Version B.07.04, Agilent Technologies, USA) with an integrated Maestro software (Version 1.4.36.16, GERSTEL GmbH, Mülheim a. d. Ruhr, Germany) was used for data acquisition and instrument control. Data analysis was carried out with MassHunter Workstation Software QQQ Quantitative Analysis (Version B.07.01 SP1, Agilent Technologies, USA).

### Method validation

The validation procedure included the assessment of linearity, matrix effects, limits of detection and quantitation as well as intraday and interday precision [52]. Calibration curves were derived from plotting the relative response (RR) against the concentrations of the calibration standards. The relative response was calculated by dividing the response of the analyte by the response of the corresponding isotopically labeled standard. Least square linear regression was carried out using the software GraphPad Prism (Version 8.3, San Diego, CA). Matrix effects (ME) were investigated by comparing the slopes of the calibration curves prepared in pure solvent and prepared in extracts of the different matrices (matrix-matched calibrations) according to Eq. (1) [52].

1 $$ \mathrm{ME}\ \left[\%\right]=\left(\ \frac{\mathrm{slope}\ \mathrm{matrix}-\mathrm{matched}\ \mathrm{standards}}{\mathrm{slope}\ \mathrm{standards}\ \mathrm{in}\ \mathrm{solvent}\ }\ \right)\ast 100\% $$

Limits of detection (LOD) and limits of quantitation (LOQ) were determined from liver blank samples. LOD was calculated by 3.3 times the standard deviation of the relative response of the blank divided by the slope of the calibration curve, whereas LOQ was calculated by 10 times the standard deviation of the relative response of the blank divided by the slope of the calibration curve [53]. The standard deviation of the relative responses of the blank was derived by injection of neat matrix extracts fortified with a solution containing the isotopically labeled internal standard mixture.

Intraday (*n* = 10) and interday (*n* = 3) precisions were determined at two different concentration levels (20 pg μL^−1^ and 500 pg μL^−1^) and expressed as relative standard deviation (RSD%). In case of testing the intraday precision, 10 replicates of the two concentration levels were injected consecutively on the same day. Three injections on four different days represented the interday precision. Potential carryover of chemicals was checked by injecting two EA blanks without matrix after every series.

## Results and discussion

### Determination of the total lipid content in liver, brain, adipose tissue and blood

Total lipids were determined gravimetrically after solvent extraction. Solvent and negative control blanks gave a low background with 0.05% of weight extracted, which were both used for blank subtraction. Positive controls showed good recoveries with a mean extraction efficacy of 99.8% for triolein and 99.1% for POPC. The tissues varied in lipid content from 0.3 to 91% (m_lipid_ m_tissue_^−1^) and triplicate analysis of blank subtracted and recovery corrected samples showed a low standard deviation (Table 1).

**Table 1:** Total lipid content (with standard deviation SD) and co-extracted matrix components after passive sampling with PDMS of the respective tissue

Tissue	Total lipid content m_lipid_ m_tissue_^−1^ (SD) [g_lipid_ kg_tissue_^−1^]	Co-extractives in PDMS m_coextractives_ m_PDMS_^−1^ [mg_coextractives_/g_PDMS_]	PDMS used for extraction m_PDMS_ [mg_PDMS_]	Amount of co-extractives injected in GC with 1 μL injection volume of final extract with 50 μL volume [μg]
Pork liver tissue	39.7 (0.8)	6.31	125	15.8
Pork brain tissue	105.0 (0.6)	1.37	330	9.0
Pork fat tissue	809.6 (5.1)	7.71	125	19.3
Pork blood	3.1 (0.1)	0.45	400	3.7
Human liver tissue	36.2 (0.7)	6.80	125	17.0
Human brain tissue	88.4 (0.6)	1.12	330	7.4
Human adipose tissue	910.2 (5.6)	7.60	125	19.0
Human blood	5.3 (0.2)	0.50	400	4.3

### Co-extracted matrix components in PDMS

During passive sampling, not only the target analytes but also undesired lipophilic matrix was taken up by the PDMS. This led to a weight gain of the PDMS, which was monitored by weighing the PDMS before and after passive sampling. The co-extracted matrix can be attributed to lipids or lipid-like substances [23]. Different amounts of co-extractives were extracted depending on the tissue sampled. For blood, there was only 0.05% of weight gain (m_coextractives_ m_PDMS_^−1^), but in fat tissue, 0.8% lipid was co-extracted (Table 1). Accordingly, different amounts of co-extractives were injected into the GC (Table 1).

### Problems with interfering matrix components in conventional GC-MSD methods

**Fig. 1:**
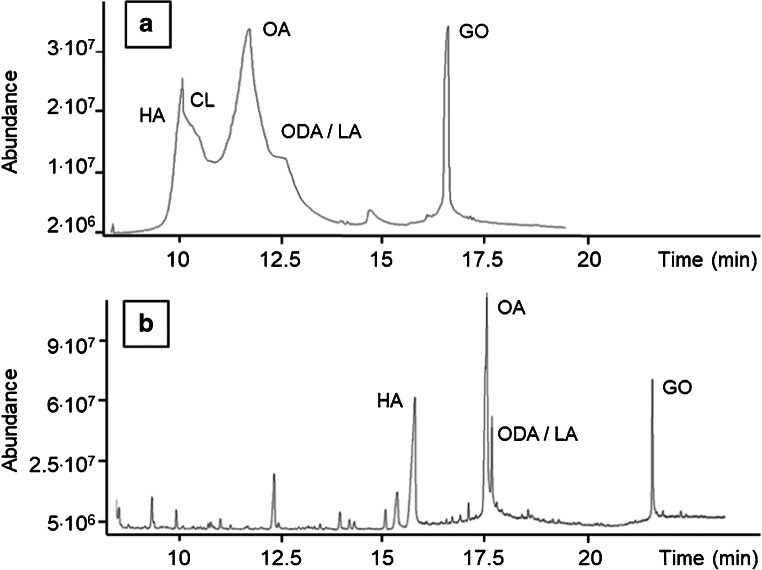
Panel **a** shows a full scan chromatogram (*m/z* 50–550) of pork liver blank matrix extract measured with GC-MSD. A full scan chromatogram (*m/z* 50–550) of pork liver blank matrix extract measured with DSI GC-MS/MS is displayed in panel **b**. The injection volume was 1 μL of PDMS extract with a total amount of 16 μg co-extractives co-injected in both measurements. Identified co-extractives in both chromatograms were hexadecanoic acid (HA), cholesterol (CL), oleic acid (OA), linoleic acid (LA) overlapping with octadecenoic acid (ODA), and glyceryl oleate (GO)

The analytical method was first tested on a single quadrupole mass spectrometer (GC-MSD) system with SSL injector (details can be found in the ESM, Section S4) where different problems caused by the co-extracted matrix occurred during method development. Figure 1 a shows a full scan chromatogram (*m/z* 50–550) of neat pork liver blank matrix extract which was measured on the GC-MSD. The co-extracted matrix components were identified using the NIST® Database. Volatile as well as non-volatile matrix components produced a high background, since the whole matrix that was present in the extract was injected. The PDMS gained 0.63% of weight during equilibrium passive sampling with liver (corresponding to 16 μg co-extractives) and most of this weight gain could be attributed to lipids. Peaks in Fig. 1 were identified as hexadecanoic acid (HA) and cholesterol (CL), oleic acid (OA), linoleic acid (LA) overlapping with octadecenoic acid (ODA), and glyceryl oleate (GO). The peak at 15 min in Fig. 1 a could potentially be octadecanal (OL), but this identification is inconsistent with the Kovats retention index. OL would be expected to elute before OA. We therefore did not label this peak. The early eluting CL peak can possibly be caused by a carryover, since according to the retention indices, CL should elute at the rear of the chromatogram.

By measuring a 50 pg μL^−1^ solution spiked on blank liver matrix in selected ion monitoring (SIM) mode, all compounds could be detected, but a raising baseline at 11 min due to co-eluting matrix components and ghost peaks were observed (ESM Fig. S1) that adversely affected quantitation of co-eluting analytes and the long-term stability of the measurements. For these reasons, this method is unsuitable for the analysis of the PDMS extracts unless clean-up is performed prior to GC analysis.

### Reduction of matrix effects by the use of DSI GC-MS/MS

To reduce the matrix effects, a GC-MS/MS system with a special injector was selected. The injection system consisted of the TDU which was connected to the CIS. The liquid sample extract was injected into the TDU at temperatures below the boiling point of the solvent. Disposable μ-vials were used, which were placed inside a TDU tube with notch. The amount of interfering matrix that was transferred onto the analytical column was reduced by this method but some volatile matrix components still entered the system. An improvement of the method with TDU (Fig. 1b) compared with the classical liquid injection method (Fig. 1a) was the partial removal of interfering non-volatile matrix components at the injection system. Only volatile matrix components including free fatty acids could be identified in the DSI samples (Fig. 1b).

During the thermodesorption cycle, non-volatile matrix components remained in the μ-vial (ESM, Fig. S6—visible lipid droplets). Volatile or semi-volatile matrix components, for example, fatty acids, were refocused in the CIS at lower temperatures before the start of the GC run together with the target analytes present in the sample extract. The TDU was operated in splitless mode, but a small amount of volatile matrix components was removed by the split vent through a forced split flow of 3 mL min^−1^. Before every sample injection, the TDU tube was exchanged. Compared with the option of combining TDU tubes with μ-vials, the use of glass wool in TDU tubes was less efficient and introduced the risk that glass wool entered the GC system. This led to generation of additional active sites in the injector port and carryover of analytes. Parameters for DSI method optimization are outlined in the ESM, Section S3.

### Method validation

Analysis for the 27 chemicals was performed in MRM mode (ESM, Fig. S2). Calibration curves of all analytes were linear (*R*^2^ > 0.994) over the measured concentration range (Table 2) and slopes with standard errors calculated by error propagation are reported in ESM (Table S4). Blanks of pure solvent, cleaned PDMS (without matrix), and liver matrix extracts showed no contamination and the results were below LOQ (ESM, Fig. S3–S5). Matrix effects (ME) for the three different tissues and blood are visualized in Fig. S7. For the assessment of matrix effects, the slopes of the different matrix-matched calibrations were compared with the slope of a calibration in EA according to Eq. (1) using the slopes listed in ESM Table S4. We considered an ME of 100% ± 20% as acceptable range (dotted lines in Fig. S7). For 19 compounds (all PCBs, all organophosphorus pesticides, ATZ, TCEP, MTC, TPP, and DDT), ME values ranging between 88 and 120% and the standard error for all four matrices were obtained (ESM, Table S5). For Irgarol (IGL), DDD, and DDE, ME values in blood matrix were up to 125% and between 74 and 122% for liver, brain, and adipose tissue. Only four chemicals, flamprop-methyl (FPM), fipronil (FPL), chlorfenapyr (CFP), and tris(2-methylphenyl) phosphate (TMPP), showed high ME values, up to 166%. For TMPP, signal suppression down to 38% was observed in liver matrix, indicating that matrix-matched calibration for quantification of these analytes must be employed ensuring an accurate quantification.

**Table 2:** Validation data obtained with the DSI GC-MS/MS method in liver extracts

Analyte	Abbreviation	LOD (pg μL^−1^)	LOQ (pg μL^−1^)	Linearity (*R*^2^)	ME (%)	Intraday precision RSD (%) (*n* = 10)		Mean relative intraday recovery (%) (*n* = 10)		Interday precision RSD (%) (*n* = 12)		Mean relative interday recovery (%) (*n* = 12)	
						20 pg μL^−1^	500 pg μL^−1^	20 pg μL^−1^	500 pg μL^−1^	20 pg μL^−1^	500 pg μL^−1^	20 pg μL^−1^	500 pg μL^−1^
Tributyl phosphate	TBP	35.2	106.6	0.994	103	n.d.	5.0	n.d.	89	n.d.	10.3	n.d.	86
Atrazine	ATZ	1.4	4.2	0.999	102	3.3	2.9	94	98	4.6	1.3	93	101
Tris(2-chloroethyl) phosphate	TCEP	7.2	21.8	0.999	105	n.d.	3.4	n.d.	105	n.d.	3.6	96	96
Diazinon	DAZ	1.8	5.3	0.999	105	2.2	0.7	103	94	1.0	0.7	103	94
2,4,4′-Trichlorobiphenyl	PCB 28	3.7	11.4	0.999	100	3.1	0.4	103	99	1.9	0.7	102	99
Chlorpyrifos-methyl	CPM	1.8	5.6	0.999	99	1.6	1.0	109	100	1.0	0.6	108	100
2,2′,5,5′-Tetrachlorobiphenyl	PCB 52	3.4	10.3	0.999	100	2.5	0.5	105	98	5.3	0.5	105	98
Metolachlor	MTC	2.0	6.0	0.999	101	1.0	0.3	102	97	0.9	0.8	103	97
Chlorpyrifos-ethyl	CPE	2.3	6.9	0.999	100	1.3	1.0	97	98	1.3	0.8	96	97
Bromophos-methyl	BOM	1.8	5.5	0.999	113	n.d.	4.3	n.d.	94	n.d.	2.2	n.d.	88
Irgarol	IGL	2.1	6.2	0.993	100	n.d.	7.5	n.d.	97	n.d.	6.8	n.d.	100
Fipronil	FPL	7.1	21.7	0.999	166	12.0	7.2	105	145	12.4	9.2	126	116
Bromophos-ethyl	BOE	1.5	4.4	0.999	119	3.2	2.9	95	94	3.6	1.6	86	87
2,2′,4,5,5′-Pentachlorobiphenyl	PCB 101	2.4	7.3	0.999	100	3.5	0.5	93	97	2.1	0.9	91	97
*p,p’*-Dichlorodiphenyldichloroethylene	DDE	2.7	8.1	0.999	74	10.5	7.9	111	91	11.5	7.0	113	99
Flamprop-methyl	FPM	4.4	13.3	0.999	146	n.d.	5.4	n.d.	103	n.d.	5.7	n.d.	91
Chlorfenapyr	CFP	29.1	88.2	0.998	135	n.d.	6.8	n.d.	107	n.d.	12.2	n.d.	85
2,3′,4,4′,5-Pentachlorobiphenyl	PCB 118	3.1	9.4	0.999	100	2.1	0.7	95	99	1.5	0.5	94	99
*p,p’*-Dichlorodiphenyldichloroethane	DDD	2.5	7.7	0.999	103	8.5	4.0	98	103	18.0	9.9	95	94
2,2′,4,4′,5,5′-Hexachlorobiphenyl	PCB 153	1.8	5.3	0.999	100	2.3	0.5	98	98	1.9	0.6	97	98
*p,p’*-Dichlorodiphenyltrichloroethane	DDT	1.0	3.0	0.999	97	2.0	1.2	95	99	9.6	1.2	96	97
2,2′,3,4,4′,5′-Hexachlorobiphenyl	PCB 138	3.3	10.1	0.999	102	2.8	1.0	99	98	1.6	0.6	99	98
Triphenyl phosphate	TPP	26.5	80.4	0.997	101	n.d.	10.3	n.d.	147	n.d.	9.6	n.d.	108
*p,p’*-Dimethoxydiphenyltrichloroethane	MOC	5.0	20.0	0.998	101	n.d.	5.4	n.d.	111	n.d.	6.7	n.d.	115
2,2′,3,4,4′,5,5′-Heptachlorobiphenyl	PCB 180	2.3	7.0	0.999	97	2.7	1.0	100	99	1.6	0.7	99	99
Tris(2-methylphenyl) phosphate	TMPP	3.0	9.1	0.999	38	7.7	6.2	122	132	15.4	12.2	151	137
2,2′,3,3′,4,4′,5,5’-Octachlorobiphenyl	PCB 194	2.5	7.6	0.999	88	4.7	4.4	96	110	6.9	6.5	122	124

For method validation, liver was chosen as most complex matrix due to the low lipid content (4%) but large quantity of co-extracted material (Table 1). LOQs ranged from 3 to 11 pg μL^−1^ for most pesticides and PCBs (Table 2). Organophosphorus flame retardants (OFRs) showed higher LOQs of 22 pg μL^−1^ (TCEP), 80 pg μL^−1^ (TPP), and 107 pg μL^−1^ (tributyl phosphate, TBP) compared with POPs like PCB 52 (LOQ = 10 pg μL^−1^) and organophosphorus pesticides like CPM (LOQ = 5.7 pg μL^−1^). Higher LOQ values for pesticides like dimethoxydiphenyltrichloroethane (MOC) and CFP of 20 and 88 pg μL^−1^ could be explained by poor fragmentation during MS/MS analysis of these compounds in addition to co-extracted matrix present in the extracts. LOD and LOQ values expressed on a lipid weight basis for comparison with other methods can be found in the ESM, Table S6.

Intraday precision was carried out by injecting 10 simultaneously prepared liver sample extracts containing 20 pg μL^−1^ (Q_low_) and 500 pg μL^−1^ (Q_high_) of all analytes. For Q_low_, the results ranged between an RSD of 1% for MTC and 12% for FPL (Table 2). For Q_high_, the best result was obtained for PCB 28 with an RSD of 0.3%, whereas the highest RSD was calculated for TPP of 10% (Table 2). Relative recoveries for intraday precision ranged from 93 to 111% for Q_low_ and from 89 to 110% for Q_high_, respectively. Interday precisions were measured by injecting three samples on four different days with a relative recovery of 86 to 126% for Q_low_ and 85 to 137% for Q_high_, respectively. RSD values were 1 to 18% for Q_low_ and 1 to 12% for Q_high_. Relative recovery was relatively high for TMPP (151% for Q_low_ and 137% for Q_high_). Q_low_ could not be quantified for the OFRs as well as for the pesticides MOC, CFP and FPL due to the high LOQs of these compounds (Table 2).

The developed method reduced matrix effects and minimized the effort for GC maintenance and sped up sample throughput because the sample extracts could be submitted to GC analysis without any additional clean-up. Nonetheless, the analysis of OFRs remains challenging. The LODs and LOQs were higher compared with other chemicals in this study and also higher deviations in relative recoveries of the spiked samples were observed. One reason for this observation could be that the OFRs tend to sorb to active sites generated by the additional glass surfaces of the TDU tubes and the μ-vials.

### Application to human samples

The validated method was applied to determine the concentrations of the 27 analytes in human tissues. PDMS and solvent blanks showed no contamination. DDT and its metabolites DDD and DDE as well as the highly chlorinated PCB congeners 138, 153 and 180 were detected in the PDMS extracts of human tissues. In blood, only DDE could be detected with a concentration below the LOQ (Table 3).

**Table 3:** Application of the method for the analysis of human tissues. Reported concentrations (C) in ng g_PDMS_^−1^ are given as mean with corresponding standard deviation (SD) from triplicate extractions

Compound	Blood C_blood_ (ng g_PDMS_^−1^)	Liver tissue C_liver_ (ng g_PDMS_^−1^)	Brain tissue C_brain_ (ng g_PDMS_^−1^)	Adipose tissue C_adipose tissue_ (ng g_PDMS_^−1^)
PCB 138	< LOD	1.5 (0.2)	1.5 (0.2)	5.2 (0.5)
PCB 153	< LOD	1.9 (0.1)	2.5 (0.2)	8.7 (0.2)
PCB 180	< LOD	1.2 (0.2)	0.8 (0.3)	5.8 (0.7)
DDE	< LOQ	90.0 (0.8)	58.0 (7.6)	115.9 (21.2)
DDD	< LOD	2.4 (0.3)	< LOD	< LOD
DDT	< LOD	2.0 (0.1)	1.2 (0.1)	2.1 (0.1)

Other non-persistent or more hydrophilic pesticides and chemicals included in the method were not detected. The measured PDMS concentrations were converted to tissue concentrations (liver, brain, and adipose tissue) normalized to the lipid content of the respective tissue (Table 4) using partition coefficients between lipid and PDMS (*K*_lipid/PDMS_) and tissue and PDMS (*K*_tissue/PDMS_) predicted with the UFZ-LSER database [54], as described in the ESM, Section S6. A comparison of the results normalized to the lipid content based on experientially determined *K*_lipid/PDMS_ from Jahnke et al. (2008) [25] is shown in the ESM, Table S9 and Fig. S8. No concentrations were calculated for the blood sample since all PDMS concentrations were below LOQ.

**Table 4:** Summary of concentrations (C) reported in ng g_lw_^−1^ (lw = lipid weight) and ng g_ww_^−1^ (ww = wet weight)

Compound	Liver tissue		Brain tissue		Adipose tissue	
	C_liver_ (ng g_lw_^−1^)	C_liver_ (ng g_ww_^−1^)	C_brain_ (ng g_lw_^−1^)	C_brain_ (ng g_ww_^−1^)	C_adipose tissue_ (ng g_lw_^−1^)	C_adipose tissue_ (ng g_ww_^−1^)
PCB 138	25.6	1.0	25.6	2.1	88.6	75.0
PCB 153	29.5	1.1	38.9	3.3	135.2	114.5
PCB 180	25.2	0.9	16.8	1.4	121.6	102.9
DDE	1761.0	64.5	1134.9	94.7	2267.8	1919.6
DDD	40.9	1.6	–	–	–	–
DDT	32.5	1.3	19.5	1.6	34.2	28.9

Previous studies investigated the burden of chlorinated POPs in several human tissues including liver [6, 7, 55], brain [7, 55], and adipose tissue [5, 6, 56]. The collected tissues were Soxhlet-extracted followed by clean-up employing GPC or sulfuric acid with acid silica gel column. Quantification was conducted either with GC-ECD (electron capture detector) or GC-MSD. Compared with our study, the results showed a similar pattern of the detected analytes with DDE as highly abundant pesticide metabolite, DDD only detected in liver as metabolic active organ, and PCB congeners 138, 153 and 180 present in all tissues as most abundant PCB congeners (comparison with results from Chu et al. [55], ESM, Fig. S9). For instance, Chu et al. [55] detected also lower chlorinated PCBs like PCB 52, PCB 101 and PCB 118 (ESM Table S10). Due to the negligible depletion criterion in passive sampling methods, only up to 5% of the analytes present in the tissue were extracted leading to concentrations below LOQ of these chemicals. In contrast to the non-depletive method, Soxhlet extraction represent a near exhaustive extraction method of the tissue enabling the analysis of environmental contaminants at much more lower trace levels than the detected concentrations of DDE, PCB 153 and PCB 180 in this study. Considering even lower concentration levels of target analytes in liver, brain and blood compared to adipose tissue, the usage of higher PDMS to sample ratio increasing the sampled amount of analytes or the application of large volume injection could compensate for this limitation.

## Conclusions

The analysis of tissue extracts obtained from equilibrium passive sampling was possible without further extract clean-up employing DSI GC-MS/MS. The advantage of the injection of the liquid extract via TDU was that non-volatile matrix components were removed offering the possibility of straight direct analysis of the sample extracts reducing time for sample preparation and costs in terms of material like solvents or longer column lifetime. The method was successfully applied to human tissues with varying lipid content. Hence, the method should be transferable to other tissues such as kidney, muscle and lung. The method was found to be suitable for POPs and non-persistent pesticides. Further optimization needs to be conducted for organophosphorus flame retardants. Analyte protectants could be used to cover active sites, which might lead to more robust results [40, 57, 58].

If passive sampling with PDMS is employed, only a small fraction of the analyte (below 5% to meet the negligible depletion criterion) is extracted, and thus, analytical detection limits might not be met. This could be the reason why lower chlorinated PCB congeners which might be present in the human tissues could not be detected in this study. But it is possible to work with depletive conditions (usage of a higher PDMS volumes in relation to the sample amount) in future studies as has been shown for blood in the present study and for sediments in the literature [59]. In these cases, the time to reach equilibrium might be longer. Another option would be to use large volume injection for the analysis of chemicals present only at trace levels to lower the detection limits in accordance with the more co-injected matrix. Furthermore, to enable a more precise determination of tissue concentrations, experimentally determined *K*_tissue/PDMS_ are required for the chemicals investigated.

## Electronic supplementary material

ESM 1 (PDF 987 kb)
