# Supplementary material for: Direct sample introduction GC-MS/MS for quantification of organic chemicals in mammalian tissues and blood extracted with polymers without clean-up
Source: Anal Bioanal Chem. 2020 Aug 15;412(26):7295–305. doi: 10.1007/s00216-020-02864-6 (PMC7497510; doi:10.1007/s00216-020-02864-6)
Supplement: Supplementary file 1 — (PDF 987 kb) [file 216_2020_2864_MOESM1_ESM.pdf]

Analytical and Bioanalytical Chemistry

Electronic Supplementary Material

Direct sample introduction GC-MS/MS for quantification of organic chemicals in mammalian tissues and blood extracted with polymers without clean-up

Andreas Baumer, Beate I. Escher, Julia Landmann, Nadin Ulrich

**Section**	**Content**	Page
S1	Additional information on the chemicals	S-3
S2	Determination of total lipid content	S-4
S3	Additional information on the DSI method	S-5
S4	Additional information on GC-MSD	S-6
S5	Additional information on GC-MS/MS	S-8
S6	Partition coefficients	S-13
S7	Comparison of tissue concentrations on a lipid weight basis (ng g_{lw}^{-1}) calculated by predicted and experimentally partition coefficients	S-15
S8	Comparison of tissue concentrations obtained in this study and Chu et al. (2003)	S-16

SECTION S1 Additional information on the chemicals

Table S1 Analytes used in this study

Analyte	Abbreviation	CAS-Number	Supplier	Lot number	Purity [%]
Tributyl phosphate	TBP	126-73-8	Alfa Aesar	D1483A	98.0
Atrazine	ATZ	1912-24-9	Cayman Chemical Company	0464014-1	Not reported
Tris(2-chloroethyl) phosphate	TCEP	115-96-8	Sigma-Aldrich	BCBS7575V	98.7
Diazinon	DAZ	333-41-5	Sigma-Aldrich	SZBC067XV	99.5
2,4,4′-Trichlorobiphenyl	PCB 28	7012-37-5	Dr. Ehrenstorfer	G151427	99.5
Chlorpyrifos-methyl	CPM	5598-13-0	Sigma-Aldrich	BCBS0018	99.8
2,2′,5,5′-Tetrachlorobiphenyl	PCB 52	35693-99-3	Dr. Ehrenstorfer	G130540	99.7
Metolachlor	MTC	51218-45-2	Dr. Ehrenstorfer	G167487	98.5
Chlorpyrifos-ethyl	CPE	2921-88-2	Sigma-Aldrich	BCBS2937V	99.8
Bromophos-methyl	BOM	2104-96-3	Sigma-Aldrich	SZBD333XV	99.5
Irgarol	IGL	28159-98-0	Fluka	SZBB265XV	98.4
Fipronil	FPL	120068-37-3	Fluka	SZBD198XV	98.6
Bromophos-ethyl	BOE	4824-78-6	Sigma-Aldrich	SZBF177XV	97.6
2,2′,4,5,5′-Pentachlorobiphenyl	PCB 101	37680-73-2	Dr. Ehrenstorfer	G164246	98.5
p,p′-Dichlorodiphenyldichloroethylene	DDE	72-55-9	Sigma-Aldrich	BCBS5816V	99.8
Flamprop-methyl	FPM	52756-25-9	Dr. Ehrenstorfer	G159922	98.9
Chlorfenapyr	CFP	122453-73-0	Sigma-Aldrich	BCBT8625	99.2
2,3',4,4',5-Pentachlorobiphenyl	PCB 118	31508-00-6	Dr. Ehrenstorfer	G151427	99.5
p,p′-Dichlorodiphenyldichloroethane	DDD	72-54-8	Sigma-Aldrich	BCBS3969V	99.9
2,2′,4,4′,5,5′-Hexachlorobiphenyl	PCB 153	35065-27-1	Dr. Ehrenstorfer	G139987	98.8
p,p′-Dichlorodiphenyltrichloroethane	DDT	50-29-3	Sigma-Aldrich	BCBS5338V	98.0
2,2′,3,4,4′,5′-Hexachlorobiphenyl	PCB 138	35065-28-2	Dr. Ehrenstorfer	G133150	99.4
Triphenyl phosphate	TPP	115-86-6	Supelco	MKBX5611V	Not reported
p,p′-Dimethoxydiphenyltrichloroethane	MOC	72-43-5	Dr. Ehrenstorfer	128313	99.0
2,2′,3,4,4′,5,5′-Heptachlorobiphenyl	PCB 180	35065-29-3	Dr. Ehrenstorfer	G164639	97.09
Tris(2-methylphenyl) phosphate	TMPP	78-30-8	Fluka	BCBP8611V	97.0
2,2',3,3',4,4',5,5'-Octachlorobiphenyl	PCB 194	35694-08-7	Dr. Ehrenstorfer	G126420	99.6

Table S2 Retention times (t_R), MRM transitions of quantifier and qualifier ions, and their collision energies (CE) for each analyte

Peak number	Analyte	Retention time t_R [min]	Quantifier ion (*m/z*)			CE (eV)	Qualifier ion (*m/z*)			CE (eV)
1	TBP	10.74	155.1	→	99.0	10	211.1	→	99.0	10
2	ATZ	12.45	199.8	→	104.0	20	214.9	→	200.1	5
3	TCEP	12.61	248.9	→	125.0	10	248.9	→	62.9	20
4	DAZ	13.33	304.0	→	179.1	10	198.8	→	93.0	20
5	PCB 28	14.57	255.7	→	186.0	30	185.8	→	151.0	20
6	CPM	14.87	285.7	→	93.0	25	285.7	→	93.0	25
7	PCB 52	15.76	291.7	→	257.0	10	291.7	→	220.0	35
8	MTC	16.39	237.9	→	162.1	10	161.8	→	91.0	40
9	CPE	16.53	313.8	→	257.9	10	313.8	→	286.0	10
10	BOM	16.92	330.8	→	315.9	20	330.8	→	93.0	35
11	IGL	17.40	181.9	→	109.1	10	237.8	→	182.0	10
12	FPL	17.44	366.8	→	213.0	40	366.8	→	255.0	20
13	BOE	17.71	358.8	→	302.9	15	330.8	→	302.9	10
14	PCB 101	17.76	325.7	→	256.0	35	325.7	→	290.9	10
15	DDE	18.19	245.7	→	176.0	35	317.7	→	246.0	20
16	FPM	18.31	276.4	→	105.0	10	276.4	→	76.9	40
17	CFP	18.57	327.8	→	247.2	15	362.9	→	246.9	35
18	PCB 118	18.67	325.6	→	256.0	35	253.7	→	219.0	20
19	DDD	18.79	234.8	→	165.1	30	234.8	→	199.0	20
20	PCB 153	19.00	359.6	→	289.9	35	359.6	→	324.9	15
21	DDT	19.35	234.8	→	165.1	20	234.8	→	199.0	20
22	PCB 138	19.42	359.7	→	289.9	35	359.7	→	324.9	10
23	TPP	19.60	214.8	→	168.0	20	232.8	→	168.1	30
24	MOC	20.26	273.8	→	239.1	10	226.8	→	140.9	35
25	PCB 180	20.50	393.7	→	323.8	35	393.7	→	358.9	10
26	TMPP	21.07	368.1	→	181.1	10	276.8	→	179.0	10
27	PCB 194	21.93	429.7	→	359.9	40	429.7	→	394.9	15

SECTION S2 Determination of total lipid content

Total lipid content was gravimetrically determined by employing a modified solvent extraction method after Smedes [1]. 50 to 500 mg of tissue were extracted in triplicates employing a mixture of water, CH and IPA (1.47 mL water, 1.3 mL of CH and 1 mL of IPA) in glass extraction vials and vortexed for 30 s. After centrifugation at 4000 rpm for 5 min, the upper CH phase was collected in a pre-weighted collection vial. Extraction was repeated three times by adding 1.13 mL of CH and 0.175 mL of IPA after each cycle. The combined solvent extracts were blown down under a gentle stream of nitrogen and further dried in a desiccator overnight and weighed in a microbalance (METTLER TOLEDO, Gießen, Germany). Total lipid content was determined gravimetrically and was corrected to negative and positive control, where bovine serum albumin (BSA) served as negative and triolein and POPC as positive controls, respectively. Additionally, a method blank containing water and extraction solvents without sample matrix was included in each batch of extraction and was treated as the samples and controls in order to exclude an extraction of any material from the used glassware and solvents caused by potential contamination.

SECTION S3 Additional information on the DSI method

TDU maintenance

Pre-conditioning of the empty thermal desorption liners with notch (for use with µ-vials) was achieved following the vendors protocol by covering the liners with a solution of DCM : MeOH (1:1 V/V) for at least 2 h. The tubes were retrieved from the solvent mixture followed by a thermal bake-out at 280 °C overnight. µ-vials, which were in direct contact with sample matrix, were cleaned by sonication with solvents of different polarities (MeOH, EA and CH) for 5 min before the pre-conditioning step described for thermal desorption liners above. Every septum, which is located inside the transport adapters, allowing liquid injection in thermal desorption tubes, was replaced at least every 40 injections.

DSI method development

The DSI method was optimized in terms of different parameters: (1) TDU heating and hold time, (2) CIS cooling temperature and time, (3) CIS temperature hold time, (4) usage of different TDU tubes (tubes with notch together with a µ-vial or tubes with frit and glass wool), (5) injection speed of solvent extracts and (6) vent flow inside the TDU. The resulted peak heights were compared and only one parameter was changed every run.

SECTION S4 Additional information on GC-MSD

Experimental method with GC-MSD

Preliminary experiments were carried out using a GC-MSD system. A GC 6890 (Agilent Technologies, USA) was coupled with a 5973 Single Quadrupole MS (Agilent Technologies, USA), which was operated in EI mode at 70 eV. Measurements were carried out using selected ion monitoring (SIM) with two ions for each compound as shown in Table S3. 1 µL sample extract was injected in splitless mode into the SSL, which was kept at 250 °C. Chromatographic separation was performed on a DB 5-MS UI® capillary column (30 m length, 0.25 µm i.d., 0.25 µm film thickness, J&W Scientific, USA). The oven was programmed as follows: 60 °C (1 min) to 210 °C at 30 °C min^{-1} (1 min), to 260 °C at 10 °C min^{-1} (3 min) and finally to 300 °C at 40 °C min^{-1} (3 min) which resulted in a total run time of 19 min. Helium (6.0 purity) was used as carrier gas in constant flow mode at 1.2 mL min^{-1} and the solvent delay was set to 6.0 min. The MS transfer line was kept at 250 °C, the ion source at 230 °C and the quadrupole at 150 °C. MS ChemStation software (Agilent Technologies, USA) was used for instrument control and data acquisition.

Table S3 Retention times (t_R) and target ions for analysis with GC-MSD (SIM mode)

Peak number	Analyte	t_R [min]	Quantifier (*m/z*)	Qualifier (*m/z*)
1	TBP	7.03	98.9	155.0
2	ATZ	7.74	200.0	215.0
3	TCEP	7.77	248.9	63.0
4	DAZ	7.95	179.1	137.0
5	CPM	8.67	285.8	124.9
6	PCB 28	8.73	255.9	186.0
7	PCB 52	9.21	291.8	219.9
8	MTC	9.33	162.1	238.0
9	CPE	9.34	196.9	313.9
10	BOM	9.71	330.8	124.9
11	FPL	9.89	366.9	212.9
12	BOE	10.37	358.8	302.8
13	PCB 101	10.55	325.8	253.9
14	DDE	11.01	245.9	317.9
15	CFP	11.22	59.0	246.9
16	PCB 118	11.60	325.8	253.9
17	DDD	11.73	234.9	165.0
18	PCB 153	11.95	359.8	289.8
19	PCB 138	12.44	359.8	289.8
20	TPP	12.72	326.0	170.0
21	PCB 180	13.87	393.7	323.8
22	MOC	13.55	227.0	-
23	PCB 194	16.01	429.7	357.8

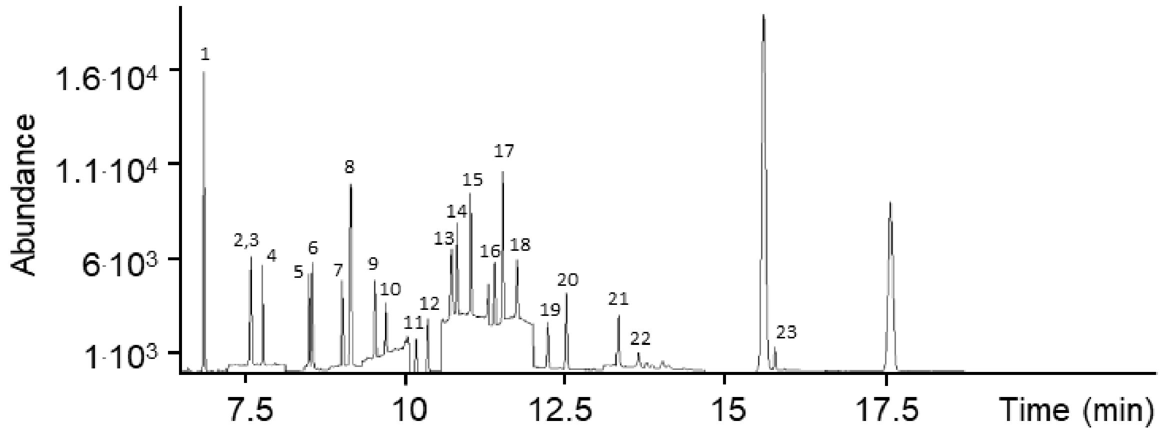

Fig. S1 Liver matrix extract containing 50 pg µL $^{-1}$ analyte solution in single ion monitoring (SIM) mode measured with GC-MSD. Peak numbers refer to elution order as shown in Table S3

SECTION S5 Additional information on GC-MS/MS

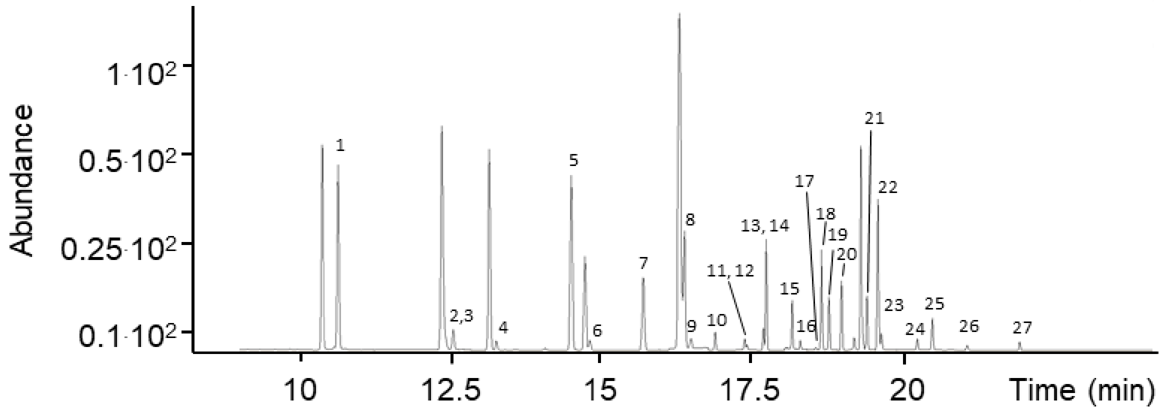

Fig. S2 Liver matrix extract containing 20 pg µL $^{-1}$ analyte and internal standard solution in MRM mode measured with DSI GC-MS/MS. Peak numbers refer to elution order as shown in Table S2

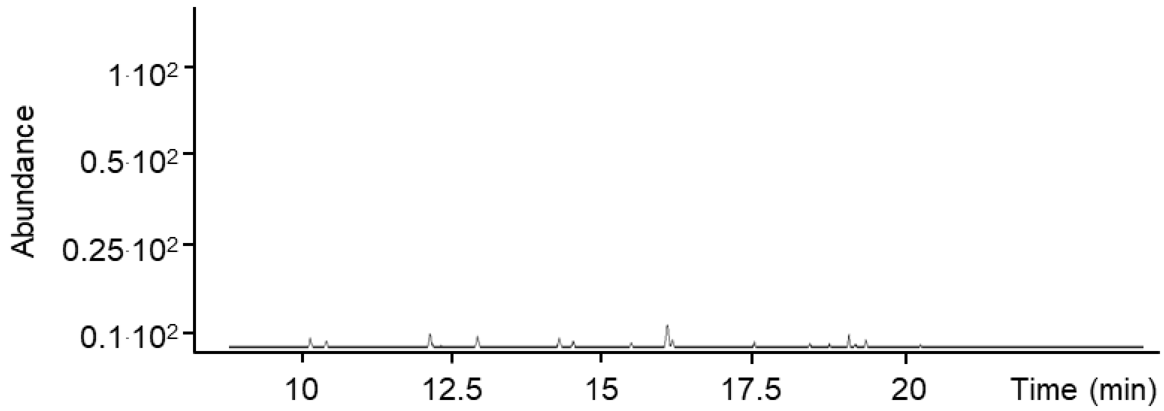

Fig. S3 Liver blank matrix extract without internal standard solution in MRM mode measured with DSI GC-MS/MS

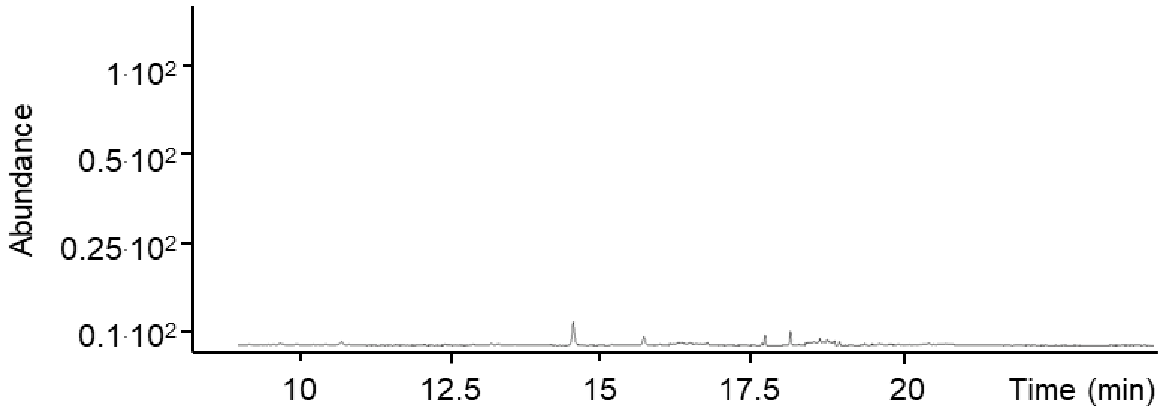

Fig. S4 Solvent blank without internal standard solution in MRM mode measured with DSI GC-MS/MS

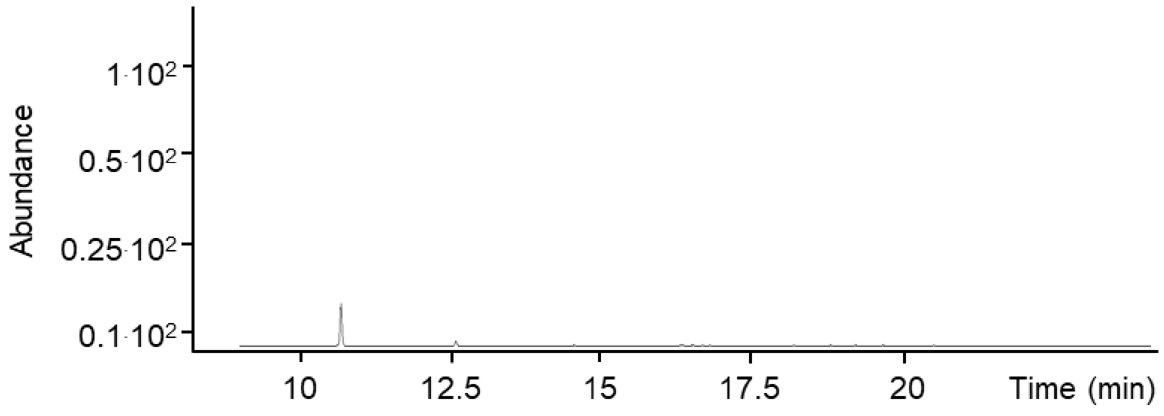

Fig. S5 PDMS blank without internal standard solution in MRM mode measured with DSI GC-MS/MS

Table S4 Comparison of slopes for calibration curves in ethylacetate and matrix-matched calibrations with consideration of errors on the slopes

Analyte	Ethylacetate		Liver tissue		Brain tissue		Adipose tissue		Blood	
	Slope	se slope	Slope	se slope	Slope	se slope	Slope	se slope	Slope	se slope
TBP	6.36E-03	9.80E-05	6.52E-03	2.32E-04	6.23E-03	2.20E-04	6.26E-03	2.84E-04	6.36E-03	9.66E-05
ATZ	4.38E-03	9.15E-05	4.46E-03	6.39E-05	4.40E-03	8.01E-05	4.26E-03	8.45E-05	4.50E-03	9.10E-05
TCEP	1.59E-03	1.85E-05	1.66E-03	1.20E-05	1.55E-03	2.52E-05	1.71E-03	2.73E-05	1.53E-03	6.25E-06
DAZ	1.49E-03	7.91E-06	1.56E-03	2.03E-06	1.51E-03	8.94E-06	1.50E-03	6.13E-06	1.55E-03	8.09E-06
PCB 28	2.01E-02	6.21E-05	2.01E-02	9.19E-05	1.99E-02	7.42E-05	2.00E-02	1.06E-04	2.00E-02	8.18E-05
CPM	5.64E-03	2.20E-05	5.56E-03	1.05E-05	5.58E-03	2.13E-05	5.71E-03	2.34E-05	5.81E-03	1.06E-05
PCB 52	1.17E-02	5.82E-05	1.17E-02	4.02E-05	1.18E-02	3.14E-05	1.16E-02	5.50E-05	1.18E-02	2.16E-05
MTC	4.55E-03	1.31E-05	4.62E-03	1.27E-05	4.51E-03	3.35E-05	4.55E-03	2.38E-05	4.68E-03	1.55E-05
CPE	4.00E-03	1.24E-05	4.00E-03	4.08E-05	4.09E-03	4.43E-05	4.00E-03	5.10E-06	4.21E-03	1.39E-05
BOM	5.09E-03	3.93E-05	5.71E-03	8.74E-05	5.62E-03	3.14E-05	5.03E-03	7.02E-06	5.59E-03	3.65E-05
IGL	1.14E-02	3.41E-05	1.14E-02	4.52E-04	1.13E-02	2.96E-04	1.16E-02	1.86E-04	1.42E-02	2.81E-04
FPL	2.13E-04	7.31E-06	3.50E-04	2.25E-06	2.69E-04	9.44E-07	3.24E-04	5.00E-06	2.81E-04	1.91E-06
BOE	6.42E-03	3.32E-05	7.61E-03	3.66E-05	6.44E-03	9.93E-05	6.00E-03	7.07E-06	7.47E-03	2.62E-05
PCB 101	2.13E-02	7.96E-05	2.12E-02	1.46E-04	2.17E-02	1.52E-04	2.11E-02	9.35E-05	2.18E-02	2.68E-05
DDE	9.73E-03	2.93E-04	7.22E-03	7.10E-05	7.53E-03	3.35E-05	8.74E-03	5.45E-05	8.89E-03	3.98E-05
FPM	3.97E-04	7.62E-06	5.78E-04	5.08E-06	4.97E-04	3.35E-06	4.51E-04	2.66E-06	6.26E-04	5.52E-06
CFP	4.80E-05	8.50E-07	6.36E-05	1.15E-06	5.56E-05	5.92E-07	4.89E-05	5.15E-07	7.58E-05	9.57E-07
PCB 118	2.43E-02	1.14E-04	2.43E-02	1.29E-04	2.40E-02	8.97E-05	2.43E-02	9.46E-05	2.44E-02	1.76E-04
DDD	9.68E-03	7.53E-05	9.93E-03	6.99E-05	9.91E-03	1.31E-04	1.14E-02	6.32E-05	1.18E-02	1.60E-04
PCB 153	2.11E-02	7.05E-05	2.10E-02	9.50E-05	2.09E-02	4.70E-05	2.08E-02	9.37E-05	2.08E-02	1.98E-04
DDT	2.65E-03	2.13E-05	2.58E-03	1.50E-05	2.64E-03	1.21E-05	2.60E-03	1.22E-05	2.78E-03	3.25E-05
PCB 138	2.16E-02	1.41E-04	2.20E-02	7.27E-05	2.20E-02	7.14E-05	2.17E-02	1.50E-04	2.24E-02	2.74E-04
TPP	2.93E-03	8.77E-05	2.70E-03	7.21E-05	2.97E-03	2.81E-04	3.37E-03	1.50E-04	3.20E-03	4.91E-05
MOC	8.94E-05	1.40E-06	8.94E-05	2.16E-06	8.21E-05	6.56E-07	1.33E-04	1.44E-06	7.36E-05	1.01E-06
PCB 180	1.98E-02	5.32E-05	1.92E-02	1.11E-04	1.94E-02	9.90E-05	1.92E-02	1.09E-04	2.13E-02	1.29E-04
TMPP	4.60E-03	8.55E-05	1.76E-03	5.33E-06	1.82E-03	2.57E-05	2.34E-03	2.14E-05	3.08E-03	5.73E-05
PCB 194	9.51E-03	2.44E-05	8.34E-03	6.41E-05	8.52E-03	1.75E-04	1.07E-02	5.59E-05	8.40E-03	2.52E-05

Table S5 Comparison of matrix effects (ME) with consideration of errors on the calculated ME

Analyte	Liver tissue		Brain tissue		Adipose tissue		Blood	
	ME (%)	se ME (%)	ME (%)	se ME (%)	ME (%)	se ME (%)	ME (%)	se ME (%)
TBP	103%	5.2%	98%	5.0%	98%	6.0%	100%	3.1%
ATZ	102%	3.6%	100%	3.9%	97%	4.0%	103%	4.2%
TCEP	105%	2.0%	98%	2.7%	108%	3.0%	96%	1.5%
DAZ	105%	0.7%	101%	1.1%	100%	0.9%	104%	1.1%
PCB 28	100%	0.8%	99%	0.7%	99%	0.8%	99%	0.7%
CPM	99%	0.6%	99%	0.8%	101%	0.8%	103%	0.6%
PCB 52	100%	0.8%	101%	0.8%	99%	1.0%	101%	0.7%
MTC	101%	0.6%	99%	1.0%	100%	0.8%	103%	0.6%
CPE	100%	1.3%	102%	1.4%	100%	0.4%	105%	0.7%
BOM	113%	2.6%	111%	1.5%	99%	0.9%	110%	1.6%
IGL	100%	4.2%	99%	2.9%	101%	1.9%	125%	2.8%
FPL	166%	6.7%	127%	4.8%	153%	7.6%	132%	5.4%
BOE	119%	1.2%	101%	2.1%	94%	0.6%	116%	1.0%
PCB 101	100%	1.1%	102%	1.1%	99%	0.8%	103%	0.5%
DDE	74%	3.0%	77%	2.7%	90%	3.3%	91%	3.2%
FPM	146%	4.1%	126%	3.3%	114%	2.9%	158%	4.4%
CFP	135%	4.7%	116%	3.3%	104%	2.9%	159%	4.8%
PCB 118	100%	1.0%	99%	0.8%	100%	0.9%	100%	1.2%
DDD	103%	1.5%	102%	2.1%	118%	1.6%	122%	2.6%
PCB 153	100%	0.8%	99%	0.6%	99%	0.8%	99%	1.3%
DDT	97%	1.3%	99%	1.3%	98%	1.2%	105%	2.1%
PCB 138	102%	1.0%	102%	1.0%	100%	1.3%	104%	1.9%
TPP	101%	5.2%	109%	12.6%	120%	8.6%	119%	4.9%
MOC	101%	4.0%	92%	2.2%	149%	3.9%	83%	2.4%
PCB 180	97%	0.8%	98%	0.8%	97%	0.8%	108%	0.9%
TMPP	38%	0.8%	39%	1.3%	51%	1.4%	67%	2.5%
PCB 194	88%	0.9%	90%	2.1%	112%	0.9%	88%	0.5%

Table S6 Conversion of LOD and LOQ values obtained from liver extracts in pg µL^{-1} extract to ng g_{lipid}^{-1}

Analyte	Liver extract [pg µL^{-1}]		Liver extract [ng g_{lipid}^{-1}]	
	LOD	LOQ	LOD	LOQ
TBP	35.2	106.6	27.5	83.3
ATZ	1.4	4.2	1.1	3.3
TCEP	7.2	21.8	5.6	17.0
DAZ	1.8	5.3	1.4	4.1
PCB 28	3.7	11.4	2.9	8.9
CPM	1.8	5.6	1.4	4.4
PCB 52	3.4	10.3	2.7	8.0
MTC	2.0	6.0	1.6	4.7
CPE	2.3	6.9	1.8	5.4
BOM	1.8	5.5	1.4	4.3
IGL	2.1	6.2	1.6	4.8
FPL	7.1	21.7	5.5	17.0
BOE	1.5	4.4	1.2	3.4
PCB 101	2.4	7.3	1.9	5.7
DDE	2.7	8.1	2.1	6.3
FPM	4.4	13.3	3.4	10.4
CFP	29.1	88.2	22.7	68.9
PCB 118	3.1	9.4	2.4	7.3
DDD	2.5	7.7	2.0	6.0
PCB 153	1.8	5.3	1.4	4.1
DDT	1.0	3.0	0.8	2.3
PCB 138	3.3	10.1	2.6	7.9
TPP	26.5	80.4	20.7	62.8
MOC	5.0	20.0	3.9	15.6
PCB 180	2.3	7.0	1.8	5.5
TMPP	3.0	9.1	2.3	7.1
PCB 194	2.5	7.6	2.0	5.9

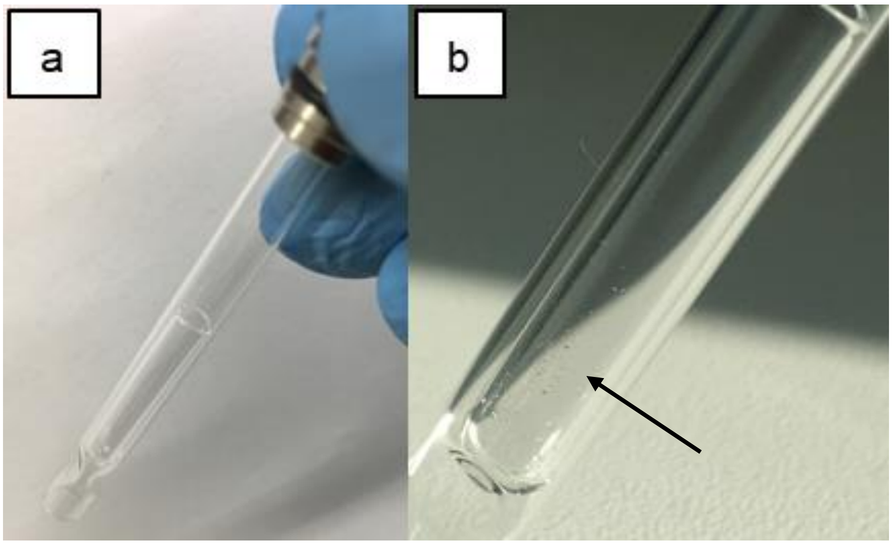

Fig-S6 TDU-tube with notch and µ-vial (a); Visible lipid droplets on the µ-vial´s glass surface after thermodesorption cycle of 1 µL of liver sample extract injected in the tube (b)

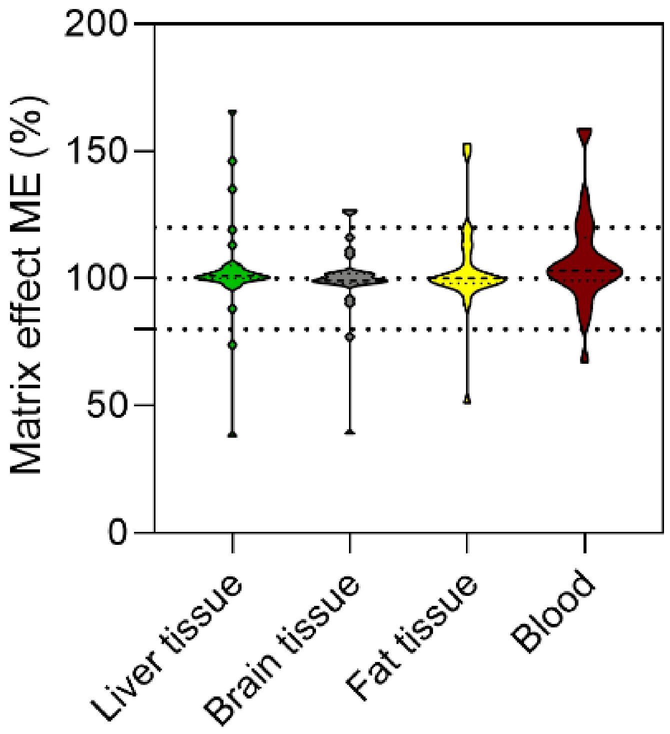

Fig. S7 Violin plot of matrix effects observed for liver, brain, fat and blood from pork

SECTION S6 Partition coefficients

Concentrations in tissues C_{tissue} were calculated by multiplying measured concentrations in PDMS C_{PDMS} (Table 3) with the partition coefficient between the tissue and PDMS ($K_{tissue/PDMS}$) according to Eq. (S1).

$$C_{tissue}=C_{PDMS}*K_{tissue/PDMS} \quad (S1)$$

Since no experimentally determined partition coefficients were available for liver and brain tissue, we calculated $K_{tissue/PDMS}$ for each tissue using the UFZ-LSER database [2].

The logarithmic PDMS-water partition coefficient (log $K_{PDMS/water}$) for each chemical detected was calculated using the equation of Stenzel et al. [3] according to Eq. (S2).

$$log\ K_{PDMS/water} = 0.37 * L – 1.55 * S – 2.85 * A – 3.84 * B + 2.37 * V + 0.46 \quad (S2)$$

The logarithmic tissue-water partition coefficient (log $K_{tissue/water}$) was obtained using the biopartitioning calculation tool embedded in the UFZ-LSER database [2]. As input parameter for the calculation of log $K_{tissue/water}$, the lipid content of the individual human tissue (Table 1) was used. Since only the total lipid was determined, no differentiation between storage and membrane lipids could be made and all the lipids were assumed to be storage lipids. The total water content for each tissue was determined, but no value for total protein content was experimentally determined. As an assumption, the tissue composition was calculated by the sum of total water and lipid content with the assignment of the missing volume fraction as protein. The input parameters which were used for the calculation are shown in Table S7. $K_{tissue/PDMS}$ can then be derived by Eq. (S3):

$$K_{tissue/PDMS}=\frac{K_{tissue/water}}{K_{PDMS/water}} \quad (S3)$$

Table S7 Input parameters for liver, brain and adipose tissue used in the biopartitioning calculation tool

Tissue	Volume fraction [%]		
	Protein	Lipid	Water
Liver	26.4	3.6	70.0
Brain	11.0	8.8	80.2
Adipose	1.5	91.0	7.5

For the calculation of concentrations present in lipids (C_{lipid}), the partition coefficient $K_{lipid/PDMS}$ is needed. Experimental values are available for different oils and ranged from 13 to 55 $g_{PDMS}\ g_{lipid}^{-1}$ (not corrected for lipid uptake in the PDMS which occurs during sampling in biota tissues) [4]. But since there are no experimental values for the $K_{tissue/PDMS}$, $K_{lipid/PDMS}$ was also calculated using the UFZ database [2]. The C_{lipid} was calculated with Eq. (S4) by multiplying the measured concentration in PDMS with the partition coefficient $K_{lipid/water}$.

$$C_{lipid}=C_{PDMS}*K_{lipid/PDMS} \quad (S4)$$

The logarithmic storage lipid-water partition coefficient (log $K_{lipid/water}$) for each chemical detected was calculated with Eq. (S5) according to Geisler et al. [5] from the UFZ-LSER database.

$$log\ K_{lipid/water} = 0.58 * L – 1.62 * S – 1.93 * A – 4.15 * B + 1.99 * V + 0.55 \quad (S5)$$

The partition coefficients used for calculation are summarized in Table S8.

Table S8 Predicted partition constants between lipid and PDMS $K_{lipid/PDMS}$ [g_{PDMS} g_{lipid}^{-1}] and between each tissue and PDMS $K_{tissue/PDMS}$ [g_{PDMS} g_{tissue}^{-1}] from UFZ-LSER database and experimentally determined partition constants between lipid and PDMS $K_{lipid/PDMS}$ [g_{PDMS} g_{lipid}^{-1}] from Jahnke et al (2008) [4]

Compound	$K_{lipid/PDMS}$ [g_{PDMS} g_{lipid}^{-1}] (LSERD)	$K_{liver/PDMS}$ [g_{PDMS} g_{liver}^{-1}]	$K_{brain/PDMS}$ [g_{PDMS} g_{brain}^{-1}]	$K_{adipose\ tissue/PDMS}$ [g_{PDMS} $g_{adipose\ tissue}^{-1}$]	$K_{lipid/PDMS}$ [g_{PDMS} g_{lipid}^{-1}] (Jahnke et al.)
DDE	19.6	0.72	1.63	16.6	19.8
DDD	17.0	0.67	1.44	14.4	51.1
DDT	16.3	0.63	1.37	13.8	32.3
PCB 138	17.0	0.65	1.43	14.4	25.5
PCB 153	15.5	0.60	1.31	13.2	27.4
PCB 180	21.0	0.79	1.76	17.7	32.2

SECTION S7 Comparison of tissue concentrations on a lipid weight basis (ng g_{lw}^{-1}) calculated by predicted and experimentally partition coefficients

Table S9 Comparison of concentrations (C) in each tissue reported in ng g_{lw}^{-1} (lw = lipid weight) derived from the calculation with $K_{lipid/PDMS}$ [g_{PDMS} g_{lipid}^{-1}] predicted from UFZ LSER database and experimentally determined $K_{lipid/PDMS}$ [g_{PDMS} g_{lipid}^{-1}] from Jahnke et al. and ratio R between concentration calculated with predicted $K_{lipid/PDMS}$ and experimental $K_{lipid/PDMS}$.

Compound	Liver tissue		Brain tissue		Adipose tissue		Ratio R
	C_{liver} (ng g_{lw}^{-1}) (LSERD)	C_{liver} (ng g_{lw}^{-1}) (Jahnke)	C_{brain} (ng g_{lw}^{-1}) (LSERD)	C_{brain} (ng g_{lw}^{-1}) (Jahnke)	$C_{adipose\ tissue}$ (ng g_{lw}^{-1}) (LSERD)	$C_{adipose\ tissue}$ (ng g_{lw}^{-1}) (Jahnke)	
DDE	1761.0	1782.0	1134.9	1148.4	2267.8	2294.8	1.0
DDD	40.9	122.6	-	-	-	-	3.0
DDT	32.5	64.6	19.5	38.8	34.2	67.8	2.0
PCB 138	25.6	38.3	25.6	38.3	88.6	132.6	1.5
PCB 153	29.5	52.1	38.9	68.5	135.2	238.4	1.8
PCB 180	25.2	38.6	16.8	25.8	121.6	186.8	1.5

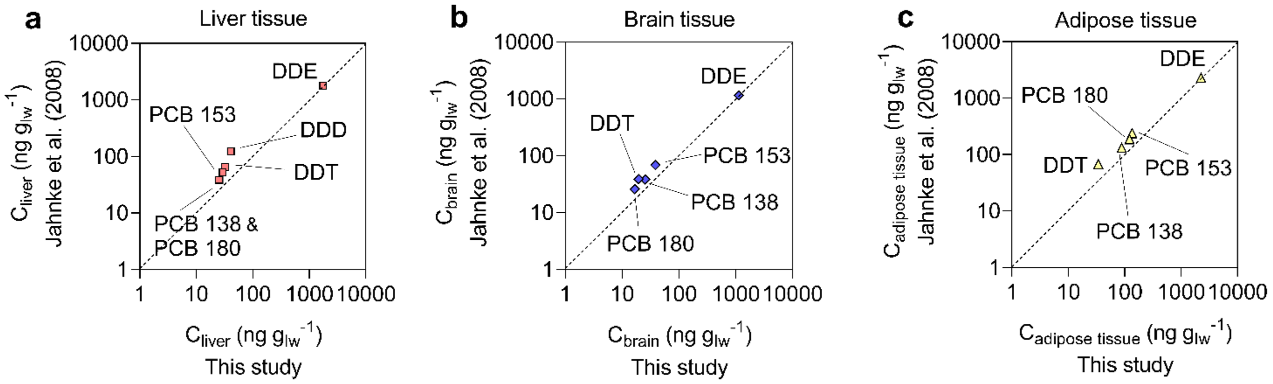

Fig. S8 Visualisation of the data from Table S9. Comparison between tissue concentrations (in units of ng g_{lw}^{-1}, lw = lipid-normalized weight) calculated by the use of predicted $K_{lipid/PDMS}$ [g_{PDMS} g_{lipid}^{-1}] from the UFZ-LSER database and experimental $K_{lipid/PDMS}$ [g_{PDMS} g_{lipid}^{-1}] from Jahnke et al. [4]. Panel a shows liver, panel b brain and panel c adipose tissue. Dashed lines indicate 1:1 relationship

SECTION S8 Comparison of tissue concentrations obtained in this study and Chu et al. (2003)

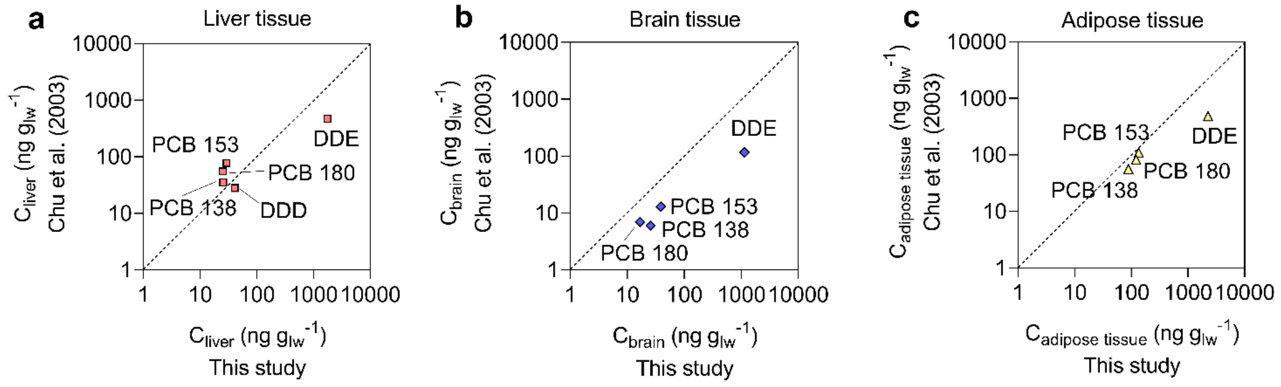

Fig. S9 Comparison between tissue concentrations (in units of ng g_{lw}^{-1}, lw = lipid-normalized weight) measured by Chu et al. [6] using Soxhlet extraction followed by extract clean-up and concentrations obtained in this study. Panel a shows liver, panel b brain and panel c adipose tissue. Dashed lines indicate 1:1 relationship

Table S10 Determination limits and mean tissue concentrations (ng g_{lw}^{-1}) of Chu et al. [6] and LOQ observed in this study expressed as ng g_{lw}^{-1}

Analyte	Chu et al. (2003)				This study
	LOD [ng g_{lipid}^{-1}]	Mean concentration in liver tissue [ng g_{lipid}^{-1}]	Mean concentration in brain tissue [ng g_{lipid}^{-1}]	Mean concentration in adipose tissue [ng g_{lipid}^{-1}]	LOQ [ng g_{lipid}^{-1}]
DDE	20	469	117	484	6
DDD	20	28	n.d.	n.d.	6
DDT	20	n.d.	n.d.	n.d.	2
PCB 52	3	5	3	3	8
PCB 101	1	4	3	4	6
PCB 118	3	17	4	20	8
PCB 138	3	35	6	56	8
PCB 153	3	78	13	109	4
PCB 180	3	55	7	82	6

n.d. not detected

References

1. Smedes F. Determination of total lipid using non-chlorinated solvents. Analyst. 1999;124(11):1711-8.
2. Ulrich N, Endo S, Brown TN, Watanabe N, Bronner G, Abraham MH, Goss KU. UFZ-LSER database v 3.2 [Internet], Leipzig, Germany, Helmholtz Centre for Environmental Research-UFZ. 2017 [accessed on 12.04.2020]. Available from http://www.ufz.de/lserd.
3. Stenzel A, Goss KU, Endo S. Determination of Polyparameter Linear Free Energy Relationship (pp-LFER) Substance Descriptors for Established and Alternative Flame Retardants. Environ Sci Technol. 2013;47(3):1399-406.
4. Jahnke A, McLachlan MS, Mayer P. Equilibrium sampling: Partitioning of organochlorine compounds from lipids into polydimethylsiloxane. Chemosphere. 2008;73(10):1575-81.
5. Geisler A, Endo S, Goss KU. Partitioning of Organic Chemicals to Storage Lipids: Elucidating the Dependence on Fatty Acid Composition and Temperature. Environ Sci Technol. 2012;46(17):9519-24.
6. Chu SG, Covaci A, Jacobs W, Haraguchi K, Schepens P. Distribution of methyl sulfone metabolites of polychlorinated biphenyls and p,p '-DDE in human tissues. Environ Health Perspect. 2003;111(9):1222-7.
